# Quality Characteristics and Consumer Acceptance of High-Moisture Mozzarella Obtained from Heat-Treated Goat Milk

**DOI:** 10.3390/foods10040833

**Published:** 2021-04-11

**Authors:** Michele Faccia, Giuseppe Gambacorta, Antonella Pasqualone, Carmine Summo, Francesco Caponio

**Affiliations:** Department of Soil, Plant and Food Science, University of Bari, Via Amendola 165/A, 70126 Bari, Italy; giuseppe.gambacorta@uniba.it (G.G.); antonella.pasqualone@uniba.it (A.P.); carmine.summo@uniba.it (C.S.); francesco.caponio@uniba.it (F.C.)

**Keywords:** mozzarella, goat milk, heat-treatment, acidification, chemical characteristics, VOC, consumer acceptance

## Abstract

High-moisture mozzarella is a pasta filata cheese manufactured from cow or buffalo milk that has spread all over the world. Its manufacturing from the milk of small ruminants (goat and sheep) has been recently proposed to innovate this ailing sector. Previously, a protocol was reported for making goat mozzarella from unpasteurized milk but, according to legislation, the microbiological safety of raw milk fresh cheeses is not guaranteed. In the present research, two new protocols were tested for producing mozzarella from pasteurized milk prepared by two different low-temperature long-time treatments (67 °C or 63 °C × 30 min). The obtained cheeses were subjected to physical–chemical and microbiological analyses and to consumer testing. The results showed that the heat treatments caused longer coagulation times than those reported in the literature, despite pre-acidification (at pH 5.93 or 6.35) having been performed to counterbalance the expected worsening of the coagulation aptitude. The obtained products showed differences in the chemical composition, texture, proteolysis, and lipolysis. Both pasteurization and pre-acidification played a role in determining these variations. Consumer testing indicated that mozzarella obtained from milk heated at the lower temperature and coagulated at a higher pH reached a good level of appreciation (62%).

## 1. Introduction

Mozzarella belongs to the family of “pasta filata” cheeses and is one of the most produced cheeses worldwide, with a global production volume over 3,000,000 tons [1]. It is manufactured at two different levels of moisture content: the low-moisture type is mostly used as an ingredient for topping pizza; the high-moisture type (HMM) is a fresh table cheese. For long time, HMM has represented a very small part of the total production volume (mostly marketed in Italy), but in the last two decades it has spread almost everywhere, and the amounts produced correspond to about 15% of the total [1]. At the time of its invention (13th century in Southern Italy) [2], mozzarella was made from buffalo milk by spontaneous lactic fermentation due to indigenous microflora. Nowadays, bovine milk has become the primary raw matter used, and the continuous increase in demand has pushed manufacturers towards industrialization and mechanization for reducing the production costs. This has been achieved by means of new manufacturing protocols based on the application of coadjuvants and commercial starters for acidification and on the use of standardized milk deriving from livestock intensive farming systems [3]. The result of this “modernization process” has been that, from being a traditional cheese, mozzarella has transformed into a food commodity that is marketed based on economic convenience. In Italy, efforts for preserving the traditional product and helping small producers to survive such an unsustainable competition have led to obtain two EU PDO (Protected Designation of Origin) acknowledgements (*Mozzarella di Bufala Campana* from buffalo milk, and *Mozzarella di Gioia del Colle* from cow milk) [4,5]. In addition to this, there is interest in producing the cheese from small ruminants’ milk (sheep and goat), which still represent an affordable market niche for small dairy enterprisers. Several scientific articles have dealt with the development of suitable protocols to be applied to these milks, but they mostly focused on the low-moisture type [6,7,8,9]. Making HMM (around 60% moisture) from sheep and goat milk is difficult, since the acidified curds that are obtained present poor stretchability and the cheese tends to harden after stretching. In a previous work, the first technological scheme for manufacturing the cheese from sheep and goat milk has been reported, based on a combination of direct acidification and lactic fermentation [10]; more recently, Tripaldi et al. [11] prepared sheep HMM by only lactic fermentation with two different types of selected starters. Basically, both investigations focused on technology and safety of the products and did not deal with the biochemical changes during storage nor with consumer acceptance.

Nowadays, goat dairy products are gaining increasing interest related to their nutritive values and positive health benefits [12]. Unfortunately, these are more susceptible to be contaminated by foodborne pathogens than the bovine counterparts. In addition, a meta-analysis published by Gonzales-Barron et al. [13] demonstrated that the foodborne pathogens incidence in goat cheeses is particularly high, with *Listeria monocytogenes* and *Staphylococcus aureus* reported as the most pathogenic agents. The investigations on goat and sheep HMM mentioned above employed raw milk, but none of the pathogens listed in the European Union regulation were detected. Nevertheless, it is known that the thermal treatment applied during the stretching phase cannot guarantee safety, unless the temperature of the curd is maintained above 80 °C for a suitable time [14]. Moreover, Tirloni et al. [15] reported that the chemical characteristics of mozzarella are insufficient to prevent the growth of *L. monocytogenes*. Since the stretching temperatures applied by Faccia et al. [10] for making goat mozzarella were below the safety levels, a suitable protocol for preparing the cheese from pasteurized milk should be developed. Unfortunately, milk pasteurization guarantees safety but negatively affects cheesemaking, with particular reference to milk coagulation aptitude and cheese texture and flavor [16,17,18].

The present investigation aimed at obtaining high-moisture mozzarella from heat-treated goat milk, without compromising the overall quality. Two different cheesemaking protocols were tested, which differed in the intensity of the milk heat treatment and in the value of milk pH at the time of rennet addition. The obtained cheeses were characterized by physic–chemical and microbiological analyses and were subjected to consumer testing in order to evaluate acceptance.

## 2. Materials and Methods

### 2.1. Cheesemaking Protocols

**Figure 1:**
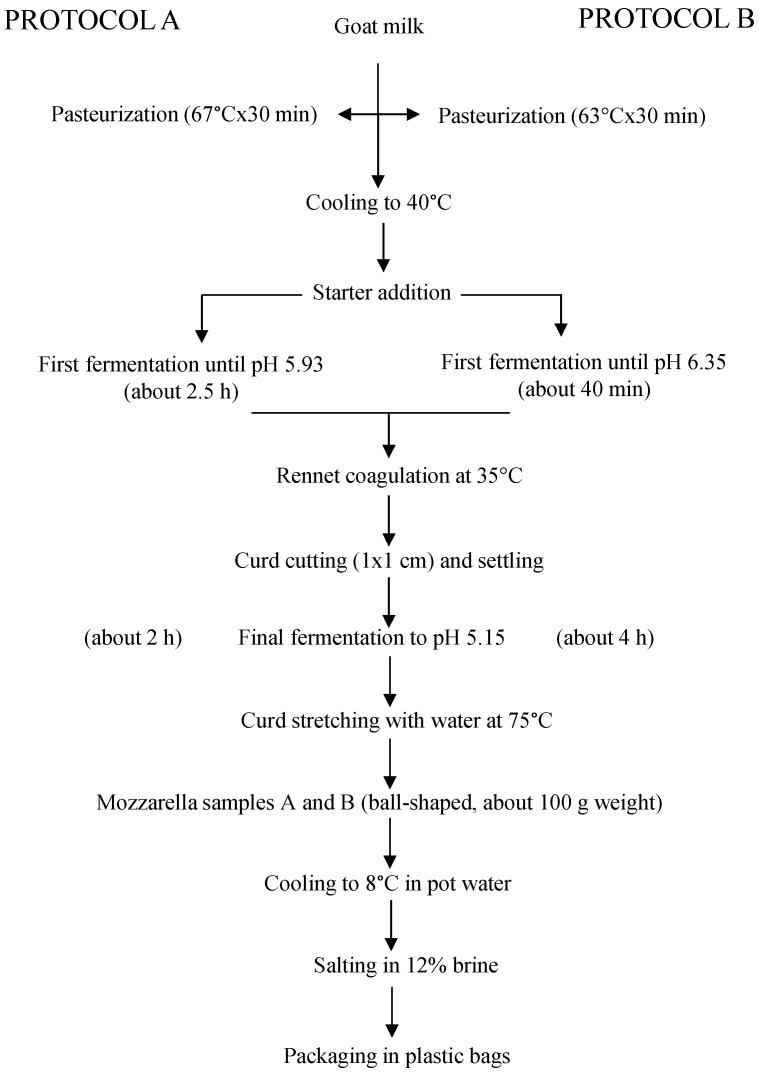
Cheesemaking protocols adopted in the experimentation.

Two different cheesemaking protocols (A and B) were applied, which differed in the intensity of milk heat treatment and value of milk pH at the time of rennet addition (67 °C × 30 min, pH 5.93 and 63 °C × 30 min, pH 6.35, respectively). The heating parameters were those most commonly applied in low-temperature long-time pasteurization, whereas the pH values were chosen to compare the one contemplated in the previously reported protocol (5.93) [10] with the one that is applied, on average, for making traditional cow mozzarella (6.35). The heat treatment was carried out in the same vat that was successively used for cheesemaking. The other applied cheesemaking parameters derived from the previous work [10], with some modifications regarding the temperature of coagulation and the rennet dose (both were slightly higher) and the type of starter (an artisanal whey culture was used instead of a commercially selected starter). The whey culture was obtained from a local dairy that produced it daily by the traditional back-slopping procedure with incubation at ambient temperature. It is known that it is an undefined mixture of autochthonous lactic acid bacteria. Each protocol was performed in duplicate, and four cheesemaking trials were performed in total. The two batches of milk used in the experimentations derived from the same farm, were taken a week from each other, and were immediately processed upon arrival at the laboratory for minimizing the differences between trials. To this aim, their gross composition and microbiological characteristics were assessed. The cheesemaking trials were performed at the Food Technology Laboratories of the Department, in a pilot plant composed of two 30-L stainless steel vats heated by indirect steam. Calf rennet (153 I.M.C.U 92% chymosin, 8% pepsin, Sacco srl, Cadorago, Italy) was added at the ratio of 20 mL L^−1^ milk for coagulation. A combined pH meter/thermometer apparatus (Crison, Barcelona, Spain) continuously controlled the temperature and pH during processing. The obtained cheeses (ball-shaped, around 100 g weight) were immediately cooled by immersion in pot water and then transferred into chilled water until the core temperature reached 8 °C (as measured by a thermometer probe). After cooling, the cheeses were weighed on a technical balance for the calculation of the cheesemaking yield, expressed as kg cheese per 100 kg milk. Then, the cheeses were salted for 10 min in a 12% brine kept at 8 °C, packaged in plastic bags immersed in water as governing liquid, and stored under refrigeration for 1 week, which is the usual shelf-life for traditional HMM. They were analyzed for physic-chemical and microbiological parameters at day 0 and 7 after production. The cheesemaking protocols applied are summarized in Figure 1.

### 2.2. Physical–Chemical and Microbiological Analyses

The fat and total protein contents of the milk were determined by the Gerber [19] and Kjeldahl [20] methods, respectively, whereas pH was measured by immersion of a pH probe (Hamilton Company, Bonaduz, Switzerland). The cheeses underwent to analysis of moisture [21], pH [22], fat [23], total protein [20], and lactose by the enzymatic method [24]. Texture profile analysis was performed by means of a Z1.0 TN texture analyzer (Zwick Roell, Ulm, Germany) equipped with a stainless-steel square probe (4 cm side) and a 1 kN load cell. Data were acquired by means of the TestXPertII v. 3.41 software (Zwick Roell, Ulm, Germany) at a frequency of 400 Hz. For each test, a mozzarella cylinder of a 2 cm diameter and 2 cm height was prepared, and the probe was moved down onto the sample surface. The conditions in the cyclic compression test were 1 mm/s probe compression rate, a 60% sample deformation in both the compressions, and a 10 s pause before the second compression. The analysis measured hardness (N), springiness (measured by the distance of the detected height during the second compression divided by the original compression distance), gumminess (N), and chewiness (N).

For the microbiological analyses, serial decimal dilutions of milk and cheese were prepared and plated on media, then the plates were incubated under suitable conditions. Basic information about the microbiological quality of milk (before and after pasteurization) was obtained by counting total mesophilic bacteria (TVC) on a Plate Count Agar incubated at 30 °C for 72 h, and total coliforms (TC) on a Violet Red Bile Lactose Agar at 37 °C for 24 h. As for the cheese, a 10 g sample was diluted in 90 mL of 2% (wt/vol) sodium citrate solution and homogenized in a Waring blender (Waring Commercial, Torrington, CT, USA). Total mesophilic bacteria were counted as reported for milk; presumptive lactobacilli on a de Man–Rogosa–Sharpe agar, pH 5.4, at 30 °C for 48 h under anaerobiosis; lactococci and streptococci on M17 agar containing 10% lactose at 37 °C for 48 h; total coliforms on Violet Red Bile Lactose Agar at 37 °C for 24 h. Yeasts and molds were counted on Yeast Extract Dextrose Chloramphenicol Agar at 30 °C for 96 h. All determinations were made in duplicate and expressed as log colony-forming units per gram of cheese. The presence of *Salmonella* spp. and *Listeria monocytogenes* in 25 g samples was assessed by the recommended reference methods [25]. All media were from Oxoid (Basingstoke, UK).

### 2.3. Proteolysis, Lipolysis, and Volatile Organic Compounds (VOC)

Primary proteolysis was investigated by polyacrylamide gel electrophoresis in the presence of urea (urea-PAGE) according to the method of Andrews [26]. The gel was stained with blue silver [27] and subjected to image analysis by using Quantity One software (BioRad, Hercules, CA, USA). Identification of the casein bands was made by comparison with the casein pattern reported in the previous paper on goat mozzarella [10].

Lipolysis was assessed by gas chromatography (GC) analysis of free fatty acids (FFA) as reported by Trani et al. [28]. In short, the cheese fat was extracted by the Folch method [29], dissolved in hexane, and added with undecanoic acid as internal standard; then, a solid phase extraction was performed using a STRATA NH2 cartridge containing amine-propylic resin (Phenomenex, Torrance, CA, USA) for eliminating the neutral lipids; finally, FFA were recovered by flushing the cartridge with diethyl-ether containing 2% formic acid. After trans-esterification with a boron trifluoride–methanol reagent, the samples were injected into a Fisons MFC800 GC (Fisons, Milan, Italy) equipped with a 60 m × 0.32 mm i.d. and 0.5 μm film thickness fused silica capillary column (Stabilwax, Restek, Bellefonte, PA, USA). The following conditions were applied: (a) oven—5 min at 170 °C, followed by heating (1 °C min^−1^) to 220 °C and held at 220 °C for 30 min; (b) carrier gas—helium 20 cm s^−1^ at 170 °C; (c) injector—250 °C, 1 μL, split 40:1; (d) detector—flame-ionization detector, 250 °C.

Finally, the volatile organic compounds (VOCs) in the headspace of the cheeses were analyzed by Solid Phase Micro Extraction (SPME)–GC Mass Spectrometry as reported in a previous paper [30]. In short, the VOCs were extracted at 37 °C for 15 min by a divinylbenzene/carboxen/polydimethylsiloxane 50/30 μm SPME fiber assembly (Supelco, Bellefonte, PA, USA), and the fiber was desorbed at 220 °C for 2 min in the injection port of a Trace 1300 GC connected to ISQ Series 3.2 SP1 mass spectrometer (Thermo Scientific, Waltham, MA, USA), operating in splitless mode. The operating conditions were: capillary column VF-WAX MS (60 m, 0.25 mm i.d. and 0.25 μm, film thickness Thermo Scientific); oven temperatures, 50 °C for 0.1 min then 13 °C min^−1^ up to 180 °C and 18 °C min^−1^ up to 220 °C with an isothermal for 1.5 min. Mass detector was set at 1700 V voltage; source temperature, 250 °C; ionization energy, 70 eV; scan range, 33–200 amu. Peak identification was performed by means of Xcalibur V2.0 Qual Browse software by matching with the NIST library reference.

### 2.4. Consumer Test

Acceptance testing of the experimental cheeses was conducted on a sample of Apulian cheese consumers (Puglia Region, Italy). They were recruited using a survey launched on the Department website and on two different Facebook pages with thousands of subscribers managed by the Apulian Section of the Italian Association of Cheese Tasters. More than 100 self-reported consumers of mozzarella, purchasing the product at least three times a month, participated to the study. All of them were invited to join two sessions (one for each type of mozzarella) that took place in different days in a reserved room at a restaurant close to the University Department. The participants were seated at tables for eight people each, where they received the samples under the guidance of a trained expert. Only fresh mozzarella samples (1 day after production) were evaluated, cut into slices weighing 25–30 g each, and served into small plastic dishes with a fork. One slice was offered to each assessor but, on request, a second slice was available. The serving temperature was 15 °C. Consumers were asked to evaluate the product using a 4-point hedonic scale from not appreciated (0) to highly appreciated (3). The responses were written on a scorecard and given to the guide. The answers were grouped according to age and sex of the assessors and expressed as percentage of total.

### 2.5. Statistical Analysis

All analyses were carried out in triplicate. The data were statistically processed by one-way ANOVA followed by Tukey’s HSD procedure at *p* < 0.05 using XLSTAT software (Addinsoft, NY, USA).

## 3. Results and Discussion

### 3.1. Physical–Chemical and Microbiological Characteristics of Milk and Cheese

The chemical and microbiological characteristics of the two batches of raw milk used in the experimentation were almost the same. The average values were (for A and B trials, respectively) 4.10–4.03% fat; 3.75–3.71% protein; 6.69–6.71 pH; 5.66–5.59 log cfu mL^−1^ TVC; 3.88–4.14 log cfu mL^−1^ TC. After pasteurization, the values of the counts decreased to 4.20–4.27 log cfu mL^−1^ TVC and < 100 cfu mL^−1^ TC. The coagulation times measured from the rennet addition to curd cutting (empirical evaluation of curd firmness) varied slightly (18 ± 0.7 min in A versus 21 ± 1.7 min in B). In both cases, it was a longer time than that reported in the previous research (12 min) [10], indicating that the heat treatment worsened the coagulation aptitude of milk. This finding does not match with the results of Calvo [31], who did not find any increase in the coagulation time of whole goat milk heated to 70 °C for 30 min. It is very likely that such discrepancy derived from the fact the milks underwent to different thermal damage since the cooling rates were different. In fact, in our experimentation, we used 30 L milk per trial, and cooling was performed by the circulation of running water into the steam jacket of the vat (this required around 30 min); in contrast, Calvo performed the experimentation on very small milk volumes (glass tubes 16 × 62 mm), and the heated samples were immediately cooled in an ice-water bath. In practical terms, our experimentation reflected the real conditions of cheesemaking in small traditional dairies. The effect of pH at the time of rennet addition was also relevant, since it allowed a shorter coagulation time in the correspondence of the most intense heat-treatment.

Table 1 shows the chemical composition of the cheeses. They differed significantly in the moisture level and, apparently, in the fat and protein content. Nevertheless, these latter two parameters were not different when calculated on a dry matter basis, suggesting that the extent of their retention from milk into cheese was not affected by the variation of the technological parameters. Of course, the higher water retention in the A samples determined a higher yield. As expected, pH decreased and lactose disappeared at the end of the storage time because of microbial fermentation. During storage, an increase in the moisture content was observed in both mozzarella samples, but it was statistically significant only for A. Water absorption in this product depends on the fact that it is stored in water, which determines a sort of osmosis due to the inequality of the chemical potential on the two sides of the membrane (the “skin” of mozzarella) [32]. Such “pseudo-osmotic” event seems to have been flavored by the softer texture of cheese A, as evidenced by the results of texture analysis (Table 2). From the data, it clearly appears that the cheeses had different characteristics at both sampling times. Mozzarella obtained from the application of the protocol A evidenced a lower level of all measured parameters with respect to that manufactured with protocol B.

**Figure 2:**
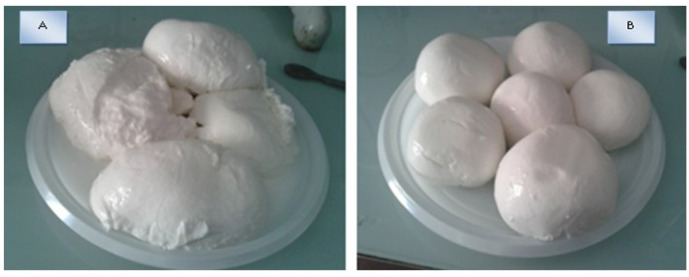
Goat mozzarella obtained with protocol (**A**,**B**) after 7 days refrigerated storage.

In particular, hardness at day 0 was roughly half the level of the other and then decreased to one third at day 7. Moreover, the values of gumminess and chewiness fell below 1 at the end of the storage time, as already evident from the appearance of the cheese, indicating that texture was compromised (Figure 2). This finding indicated that protocol A, although providing a high cheese yield, is not suitable for obtaining a cheese with long shelf-life.

The difference in the moisture level between the products (less than 3%) appears as not sufficient to justify the strong variation in texture. Rather, the softer body of cheese A should be connected to stronger calcium depletion from the casein micelle induced by the longer acidification phase in milk [33]. However, since the protein contents in the cheeses were not different, it is likely that calcium depletion did not cause a loss of casein into the whey. It should have been prevented by the high temperature at which acidification took place (38–40 °C), since dissociation of the caseins from the micelles on acidification is temperature dependent and becomes very relevant at low temperatures [34].

Table 3 shows the microbiological profiles of the cheeses. The most important difference was in the count of lactococci and streptococci, which was nearly one log cycle higher in the cheese from protocol B. It suggests a sort of inverse relationship with the incubation times of the starter into the milk before rennet coagulation and could be explained by faster growth of these LAB groups into the curd than in milk. A similar trend was observed for coliforms.

The second group in order of abundance was total mesophilic bacteria, but their counts were not significantly different between the cheeses. The same was for lactobacilli, whose relatively low count values indicated that they were not primary compounds of the autochthonous starter used. As to the hygienic quality, pathogens were absent, and the values of *Enterobacteriaceae* and coliforms were within the range commonly found in artisanal cow’s HMM [35,36]. They should derive from the autochthonous starter added, since cheesemaking was carried out at the laboratory pilot plant under good hygienic conditions.

### 3.2. Pimary Proteolysis and Lipolysis

**Figure 3:**
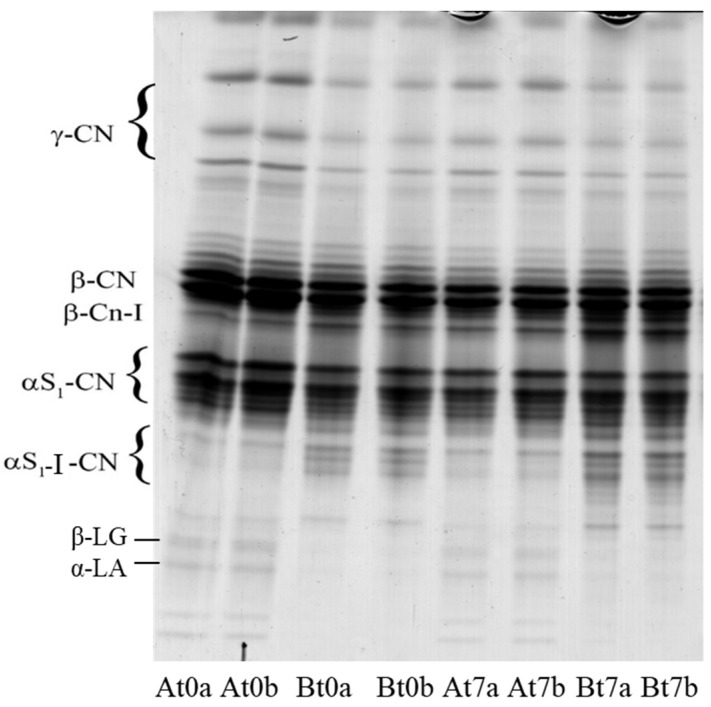
Urea-PAGE of goat mozzarella samples during storage (t); a and b are samples from two replicate trials.

The electropherogram of the samples is shown in Figure 3. Even though proteolysis was poor due to the short time from manufactuirng, some differences were detectable between cheese A and B. The former evidenced a higher intensity of the bands attributable to γ-caseins, the latter contained a higher level of αs1-I- and β-I-caseins (the primary proteolysis products of αs1- and β-casein).

It is known that the primary source for γ-caseins formation in fresh cheeses is the hydrolytic activity of plasmin (PL) towards β-casein, whereas αs1-I- and β-I-fragments are released by rennet enzymes that residuate in the curd. PL activity in cheese depends on a series of variables that influence its activation from plasminogen (PG) by the so called “plasmin system”. It is known that milk pasteurization has a differentiated effect on the compounds of PL system, which present different thermal stability. Pasteurization increases PG activation due to denaturation of the plasmin activator inhibitor, whereas stronger treatments tend to reduce PL levels [37]. In our experimentation, PG activation should have been more favoured by treatment at 67 °C × 30 min with respect to 63 °C × 30 min, causing higher formation of γ-caseins. It is likely that such formation took place in the processing phases that preceded curd stretching since the intensity of the bands did not increase during storage, suggesting poor PL activity in the cheeses. This latter aspect is worthy of specific investigation since the effect of streching on plasmin activity in HMM is unknown; however, Kiely et al. [38] reported that plasmin was inctive in low-moisture mozzarella manufactured with pasteurized cow milk. Additionally, αs1-I- and β-I-fragments at day 0 should have mostly formed in the time preceeding stretching. In fact, it has been reported that this treatment causes partial or total denaturation of residual chymosin, whose activity in mozzarella is absent or very scarce [39]. The faster accumulation of the two proteolytic fragments at day 0 in cheese B depended on the longer hold time of the curd before being stretched. In contrast to γ-caseins, they slightly increased over time in the products, suggesting that rennet enzymes were not totally denatured. The patterns also evidenced the presence of two barely visible bands in the zone below the αs1-I-fragment that should be attributed to β-lactoglobulin and α-lactalbumin [40]. These bands are good indicators of the milk heat treatments since their intensity was directly proportional to the temperature applied.

Information about lipolysis was obtained by analysis of FFA (Table 4). The patterns of the two cheeses were signifcantly different, both qualitatively and quantitatively. Overall, the total concentration was rather low, as expected in a fresh product such as mozzarella, but cheese A was less lypolized than B already at day 0 (2.14 mg vs. 4.10 mg g^−1^ fat). At this time, the only fatty acids detected in A were stearic, palmitic, and, at a very low level, oleic, whereas all fatty acids with an even number of carbon atoms from C8 to C20, plus margaric acid (C17), were detected in cheese B. As expected, the total concentration increased after 7 days, and even though the differences between the two cheeses decreased, they remained highly significant.

In general, the total amounts observed were in line with those reported by Murgia et al. [41] in Fruhe, a traditional fresh goat cheese from Sardinia (Italy); however, a correspondence was not found under the qualitative point of view. In particular, the short-chain acids lacked in mozzarella, which probably depended on the shorter storage time, but also on type of rennet used. In fact, goat mozzarella was manufactured with liquid calf rennet, whereas Fruhe is manufactured with kid rennet paste, which contains a pregastric esterase that is very specific for short-chain fatty acids [42]. What remains to be understood is the different extent of lipolysis in the two types of mozzarella. The experimental design does not allow one to make any reliable hypothesis, since some important aspects, such as the load in psychrotophic bacteria (highly lipolyic), were not investigated. The only considerations that can be made regard the activity of the endogenous lipase system of the milk. According to Collins et al. [43], pasteurization causes an average 73–95% inactivation; thus, it is likely that the different intensity of the milk heat treatment had a role in causing different rate of lipolysis in the two cheeses.

As to the role of the starter microflora in these two biochemical events, this has not been considered in the discussion since it is known that LAB are scarcely involved in primary proteolysis, and their lipolytic activity is relevant only in long ripened cheeses. Instead, they are the main agents of secondary proteolysis, which becomes relevant during ripening, giving a pivotal contribution to the flavor [44]. In the present study, the storage time of the cheeses was too short for expecting relevant secondary proteolysis; thus, this event was not investigated.

### 3.3. VOC

Overall, 35 volatile compounds were identified in the entire set of samples (Table 5). The most represented group was that of terpenoids, followed by ketones; after 7 days, acids increased and became a relevant group. The pattern was typical of a fresh cheese, and the level of terpenoids was, unusually, very high. In fact, these compounds accounted for a large part of total VOC: at day 0, they represented about two third of total in cheese A and more than 40% in cheese B; at day 7, the level approached 50%. Such a richness in terpenic compounds is not common and has been sometimes reported in milks from animals grazing for a long time in particular geographical areas on natural pastures in the flowering seasons [45,46,47,48]. The milk used in the present experimentation derived from goats that lived fully under these conditions: it was late spring at the time of milk collection, and the animals were grazing for the most part of the day on natural pastures. The most abundant terpene was by far α-pinene: its concentration was almost double in cheese A than in B and strongly decreased over time. Since the cheeses were obtained from the same milk, the difference should derive from more efficient transfer from milk into curd during coagulation, driven by lower pH. Very interestingly, the decrease in α-pinene at day 7 corresponded to an increase in limonene, and this trend was highly evident in sample B. Bioconversion of terpenes is a challenging issue that has not yet been fully understood. Several authors have reported that microbial transformation of α-pinene into limonene is possible [49,50,51].

Fatty acids appeared in the headspace of the samples only at day 7 and were more abundant in sample B, in good agreement with the trend of lipolysis reported in Table 4. In this cheese, acetic acid reached a much higher concentration than in A; it is not included in the milk tryglicerides, but is widely formed in the metabolism of heterolactic LAB and other adventitious bacteria. Its more intense formation in B matches well with the higher counts of coliforms and Enterobacteriaceae. Among the other VOC classes, the most discriminating one was that of ketones, even though the difference between the cheeses was mostly determined by acetoin and acetone, whose levels were very high (appearing as abnormal) in B cheeese at day 0 and in A cheese at day 7, respectively. As to acetoin, its formation is normally attributed to the activity of the starter LAB that, in the present experimentation, grew under different conditions. In the protocol A, about one half of the fermentation took place in milk, whereas it was entirely performed in the curd in protocol B. In the A protocol, it is likely that a great part of the water-soluble fermentative metabolites (as is acetoin) remained in the whey upon curd extraction. This should explain the general higher level of VOC in B samples at time 0, except terpenoids (which, in fact, are fat soluble). For acetone, the limited microbiological analyses performed in the present experimentation did not allow us to make sound hypothesis about its origin. However, it has been reported to be among the compounds of the volatilomes produced by strains of non-starter *Lactobacillus casei* and *Pseudomonas* of dairy origin [52,53]. Finally, it has to be highlighted that alcohols, ketones, and esters underwent a sharp decrease over time. It was not surpising, since the cheeses were stored immersed in water, and this causes mass exchange phenomenon leading the most soluble compounds being partially lost into the liquid.

### 3.4. Consumer Testing

**Figure 4:**
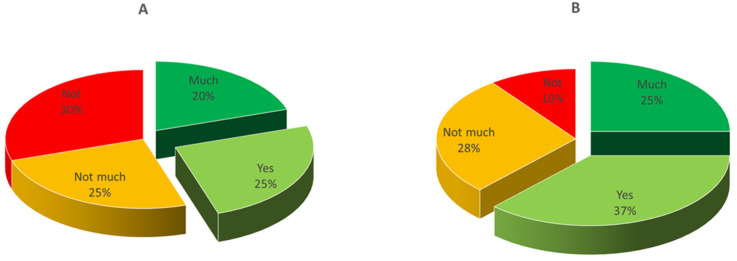
Results of consumer testing of the cheese samples from trials (**A**,**B**) at 1 day. Overall liking levels (%) are reported in the pie chart. Not = not appreciated (score 0); Not much = not much appreciated (score 1); Yes = appreciated (score 2); Much = much appreciated (score 3).

**Figure 5:**
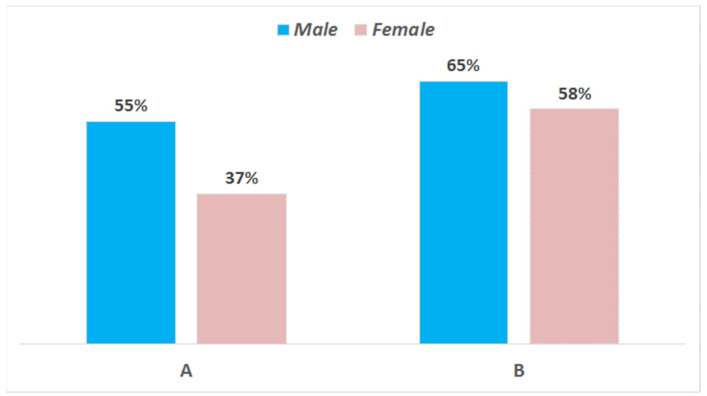
Results of consumer testing of the cheese samples from trials (**A**,**B**) at 1 day. Liking levels according to gender are reported in histograms. Percentages of positive answers (appreciated + much appreciated).

Overall, out of 122 recruited consumers, 112 showed up for the scheduled evaluation of product A and 108 for that of product B. It must be highlighted that they were not a random population, but belonged to a rather restricted group interested to cheese tasting, including students, researchers, and teachers from the University of Bari and average Apulian cheese consumers. The geographical area was chosen for logistic reasons, of course, but also because mozzarella is very popular in this region. As for gender, 45% of the tasters were male and 55% were female; regarding age, the average values for the participants in the two testing sessions were 71.7% between 20 and 30 years, 9.8% 30–40 years, 8.7% 40–50 years, and 9.8% > 50 years. Basically, it was a young-aged panel and reflected the typical profile of the Italian consumer of cow milk mozzarella. The results of testing are shown in Figure 4 and Figure 5. The cheeses from the two trials reached a different level of appreciation: only 45% of consumers appreciated cheese A (sum of appreciated + much appreciated), whereas the level of positive answers reached 62% for cheese B. Female tasters were more critical than males, even though the differences between genders was much less relevant for cheese B. Considering that the two types of samples were manufactured with the same type of milk by using the same starter and rennet, the better score for cheese B was probably connected to the better texture.

## 4. Conclusions

The present experiment demonstrated the possibility of manufacturing goat high-moisture mozzarella from low-temperature long-time pasteurized milk without any relevant technological problems. Two different protocols were developed, which gave rise to cheeses with different quality characteristics, depending on the intensity of the heat treatment and the milk pH value at which coagulation was obtained. The lower pH determined higher yield, but the product was less preservable and less appreciated by consumers, probably because the texture was too soft. In addition to this, it was found that the different combinations of the two parameters caused significant differences in primary proteolysis and lipolysis during storage. Such differences should be attributed to different levels of activation/inactivation of the enzymes involved in these biochemical events. Consumer testing revealed a good level of appreciation only for the product obtained with milk pasteurized under the milder thermal conditions and coagulated at a higher pH. The results obtained are encouraging, considering that the experimental products were innovative and that the assessors involved in the study were loyal consumers of bovine mozzarella. In fact, the risk for such a type of innovative cheese is that consumers easily reject it due to the spontaneous tendency to compare it with the product they are used to. The topic is worthy of further investigation for evaluating how the application of high-temperature short-time pasteurization could influence cheese manufacturing and quality.

## Figures and Tables

**Table 1:** Gross composition (%, except for pH, ± standard deviation) of the cheese samples from trials A and B at 0 and 7 days refrigerated storage. Values in the same column bearing different superscripts are different at *p* < 0.05; wb = on wet basis; dm = on dry matter; nd = not detected (below detection limit).

Trial	pH	Moisture	Fat (wb)	Fat (dm)	Protein (wb)	Protein (dm)	Lactose	Yield
At0	5.30 ± 0.2 ^a^	61.1 ± 1.3 ^b^	15.4 ± 1.3 ^b^	39.6 ± 3.3	17.4 ± 0.8 ^b^	44.7 ± 2.1	0.3 ± 0.1 ^a^	16.9 ± 0.7 ^a^
Bt0	5.33 ± 0.2 ^a^	58.5 ± 0.8^c^	17.2 ± 1.5 ^a^	41.4 ± 3.6	19.6 ± 1.0 ^a^	47.2 ± 2.4	0.4 ± 0.1 ^a^	14.3 ± 0.4 ^b^
At7	5.20 ± 0.2 ^b^	65.3 ± 2.0 ^a^	13.8 ± 1.9 ^b^	39.8 ± 5.5	15.0 ± 1.3 ^c^	43.2 ± 3.7	nd	-
Bt7	5.23 ± 0.2 ^b^	59.1 ± 1.7 ^b,c^	17.1 ± 1.3 ^a^	41.8 ± 3.2	18.9 ± 1.1 ^a,b^	46.2 ± 2.7	nd	-

**Table 2:** Hardness, springiness, gumminess, and chewiness of experimental goat mozzarella at 0 and 7 days refrigerated storage. X = mean value; *σ* = standard deviation. Values in the same column bearing different superscripts are different at *p* < 0.05.

Trial	Hardness		Springiness		Gumminess		Chewiness	
	X	*σ*	X	*σ*	X	*σ*	X	*σ*
At0	7.7 ^c^	0.4	0.45 ^b^	0.04	2.6 ^b^	0.3	1.9 ^b^	0.3
Bt0	15.1 ^a^	0.5	0.57 ^a^	0.03	3.3 ^a^	0.3	2.7 ^a^	0.4
At7	3.1 ^d^	0.1	0.10 ^d^	0.00	0.7 ^d^	0.0	0.4 ^d^	0.0
Bt7	9.5 ^b^	0.3	0.19 ^c^	0.00	1.5 ^c^	0.1	1.3 ^c^	0.1

**Table 3:** Microbial counts (log cfu g^−1^) in the cheese samples from trials A and B at 0 and 7 days of refrigerated storage. Values in the same row bearing different superscripts are different at *p* < 0.05.

Group	At0	Bt0	At7	Bt7
Total mesophilic	7.37 ^b^	7.47 ^b^	8.41 ^a^	8.22 ^a^
Yeasts and molds	2.90 ^b^	2.53 ^c^	3.53 ^a^	3.46 ^a^
Coliforms	3.36 ^b^	4.90 ^a^	4.35 ^a^	4.72 ^a^
*Enterobacteriaceae*	4.10 ^b^	4.23 ^b^	5.22 ^a^	5.51 ^a^
Lactobacilli	5.59 ^b^	5.34 ^b^	6.11 ^a^	6.19 ^a^
Lactococci and streptococci	8.15 ^c^	9.08 ^a^	8.44 ^b^	9.23 ^a^
*Salmonella* spp. in 25 g	Absent	Absent	Absent	Absent
*L. monocytogenes* in 25 g	Absent	Absent	Absent	Absent

**Table 4:** Free fatty acids (mg g^−1^ fat ± standard deviation) of the cheese samples from trials A and B at 0 and 7 days refrigerated storage. Values in the same row bearing different superscripts are different at *p* < 0.05.

	At0	Bt0	At7	Bt7
Butanoic (C4)	-	-	-	-
Caproic (C6)	-	-	-	-
Capyilic (C8)	-	0.02 ± 0.01 ^b^	-	0.08 ± 0.02 ^a^
Capric (C10)	-	0.08 ± 0.01 ^b^	0.17 ± 0.03 ^a^	0.21 ± 0.03 ^a^
Lauric (C12)	-	0.12 ± 0.05	-	0.15 ± 0.07
Myristic (C14)	-	0.21 ± 0.01 ^b^	0.41 ± 0.09 ^a^	0.58 ± 0.11 ^a^
Palmitic (C16)	1.30 ± 0.33 ^b^	2.04 ± 0.51 ^a^	2.10 ± 0.35 ^a^	2.56 ± 0.51 ^a^
Margaric (C17)	-	0.01 ± 0.01 ^b^	-	0.04 ± 0.01 ^a^
Stearic (C18:0)	0.61 ± 0.11 ^c^	0.90 ± 0.12 ^b^	0.96 ± 0.20 ^a, b^	1.23 ± 0.17 ^a^
Oleic (C18:1)	0.23 ± 0.05 ^c^	0.63 ± 0.21 ^b^	0.87 ± 0.14 ^b^	1.11 ± 0.16 ^a^
Linoleic (C18:2)	-	0.07 ± 0.01	-	0.11 ± 0.04
Arachidic (C20)	-	0.02 ± 0.01 ^b^	-	0.04 ± 0.01 ^a^
Total	2.14 ± 0.47 ^c^	4.10 ± 0.57 ^b^	4.51 ± 0.52 ^b^	6.11 ± 0.53 ^a^

**Table 5:** Volatile organic compounds of the cheese samples from trials A and B at 0 and 7 days of refrigerated storage (expressed as % of total peak area). X = mean; σ = standard deviation; * = different in A and B at *p* < 0.05; ** = different in A and B at *p* < 0.01.

Compounds	At0		Bt0		t0	At7		Bt7		t7
	*m*	*σ*	*m*	*σ*	*Sig*	*m*	*σ*	*m*	*σ*	*Sig*
**Acids**										
Acetic	1.8	0.4	2.6	0.5		2.6	0.6	12.5	3.4	*
Butanoic	0.0	0.0	0.0	0.0		1.9	0.3	5.7	1.0	**
Caproic	0.0	0.0	0.0	0.0		3.1	0.7	5.0	0.9	*
Caprylic	0.0	0.0	0.0	0.0		5.4	1.1	4.4	0.8	
Nonanoic	0.0	0.0	0.0	0.0		1.1	0.2	1.1	0.3	
Capric	0.0	0.0	0.0	0.0		2.2	0.5	3.9	0.9	*
Total acids	1.8	0.4	2.6	0.5		16.3	1.8	32.6	4.6	**
**Hydrocarbons**										
1,6-octadiene, 3,7-dimethyl-	1.0	0.1	0.5	0.0	*	0.7	0.1	0.4	0.1	*
Cycloheptane	0.0	0.0	0.0	0.0		0.3	0.1	0.6	0.1	*
Octane	1.1	0.2	1.7	0.2	*	0.8	0.2	0.7	0.1	
Total hydrocarbons	2.1	0.2	2.2	0.2		1.8	0.3	1.7	0.1	
**Alcohols**										
Ethanol	2.0	0.2	3.3	0.4	*	0.6	0.2	2.2	0.3	**
**Ketones**										
2-Propanone (acetone)	1.3	0.3	4.2	0.5	**	19.0	1.8	0.8	0.1	**
2-Butanone	1.8	0.5	3.6	0.7	*	0.5	0.1	1.1	0.4	*
2,3-Butanedione	2.2	0.6	3.5	0.5	*	0.5	0.1	1.5	0.3	*
2-Butanone, 3-hydroxy (acetoin)	7.8	0.7	27.0	5.4	**	4.6	0.6	3.6	0.5	
5-Hepten-2-one, 6-methyl-	0.9	0.1	1.4	0.6		0.0	0.0	0.0	0.0	*
2-Nonanone	2.0	0.5	2.0	0.1		1.4	0.5	3.6	0.7	*
Total ketones	16.0	1.6	41.7	6.8	**	26.0	2.1	10.6	0.9	**
**Aldehydes**										
Hexanal	0.0	0.0	0.0	0.0		0.7	0.4	1.7	0.5	*
Octanal	0.4	0.1	0.5	0.1		0.0	0.0	0.0	0.0	
Nonanal	1.0	0.2	2.5	0.7	*	0.0	0.0	0.0	0.0	
Decanal	0.0	0.0	0.0	0.0		1.0	0.2	1.5	0.4	*
Total aldehydes	1.4	0.2	3.0	0.7	*	1.7	0.5	3.2	0.6	*
**Aromatic compounds**										
Benzene, methyl-	3.8	0.9	1,7	0.1	*	3.0	0.6	1,1	0.4	*
Benzene, 1-methyl-2-(1-methylethyl)	1.7	0.3	1,0	0.1	*	0.0	0.0	0.0	0.0	
Benzaldehyde	0.0	0.0	00	0.0		0.3	0,1	0.5	0.1	
Total aromatic compounds	5.5	0.9	2.7	0.3	*	3.3	0.6	1.6	0.5	*
**Esters**										
Acetic acid, butyl ester	0.9	0.1	1.1	0.1		0.4	0.1	0.9	0.2	*
Acetic acid, hexyl ester	2.2	0.1	2.4	0.3		0.0	0.0	0.0	0.0	
Hexanoic acid, butyl ester	0.5	0.2	0.7	0.1		0.5	0.3	0.6	0.3	
Total esters	3.6	0.3	4.2	0.4		0.9	0.3	1.5	0.4	
**Terpenoids**										
Tricyclene	0.9	0.1	0.9	0.2		0.6	0.2	1.0	0.3	
α-Pinene	50.1	4.2	26.8	2.8	**	32.4	4.1	7.3	1.7	**
β-Pinene	7.6	0.6	5.3	0.5	*	5.1	1.6	3.0	0.6	*
Sabinene	3.1	0.6	0.5	0.1	*	2.4	0.5	0.5	0.1	*
l-Phellandrene	0.0	0.0	0.0	0.0		0.5	0.1	0.9	0.3	
Citrine	0.8	0.1	0.6	0.2		0.4	0.2	3.8	0.7	**
dl-Limonene	1.8	0.4	4.2	1.0	*	5.7	0.6	27.9	2.6	**
γ-Terpinene	0.0	0.0	0.0	0.0		0.4	0.2	0.5	0.2	
Camphene	3.4	0.4	2.0	0.3	*	2.1	0.5	1.6	0.3	
Total terpenoids	67.7	4.7	40.3	4.1	**	49.6	4.9	46.5	4.2	

## Data Availability

The data that support the findings of this study are available from the corresponding author upon reasonable request.

## References

[1] CLAL (2016). Global Pasta Filata Cheese Market Trends. https://www.clal.it/downloads/news/Dubiel-EN.pdf.

[2] (2019). Ruminantia. https://www.ruminantia.it/la-storia-della-mozzarella-di-bufala/.

[3] Natrella G., Gambacorta G., De Palo P., Maggiolino A., Faccia M. (2020). Volatile organic compounds in milk and mozzarella: Comparison between two different farming systems. Int. J. Food Sci. Technol..

[4] Council of the European Union (1996). Commission Regulation (EC) 1107/96 of 12 June 1996 on the registration of geographical indications and designations of origin under the procedure laid down in Article 17 of Council Regulation (EEC) No 2081/92. Off. J. Eur. Union.

[5] Council of the European Union (2020). Commission Implementing Regulation (EU) 2020/2018 of 9 December 2020 entering a name in the register of protected designations of origin and protected geographical indications (Mozzarella di Gioia del Colle (PDO). Off. J. Eur. Union.

[6] Pal U.K., Agnihotri M.K. (2000). Quality and shelf-life of direct acid goat milk Mozzarella cheese at refrigeration temperature. Int. J. An. Sci..

[7] Imm J.Y., Oh E.J., Han K.S., Oh S., Park Y.W., Kim S.H. (2003). Functionality and physico-chemical characteristics of bovine and caprine mozzarella cheeses during refrigerated storage. J. Dairy Sci..

[8] Shaker R.R., Attlee A., Kasi H., Osaili T.M., Al Nabulsi A.A., Ababneh A.H. (2012). Comparison of the quality of low moisture mozzarella cheese made from bovine, ovine and caprine milks. J. Food Agric. Env..

[9] Paz N.F., De Oliveira E.G., Villalva F.J., Armada M., Ramón A.N. (2017). Effect of pH at drainage on the physicochemical, textural and microstructural characteristics of mozzarella cheese from goat milk. Food Sci. Technol..

[10] Faccia M., Trani A., Gambacorta G., Loizzo P., Cassone A., Caponio F. (2015). Production technology and characterization of Fior di latte cheeses made from sheep and goat milks. J. Dairy Sci..

[11] Tripaldi C., Palocci G., Di Giovanni S., Marri N., Boselli C., Giangolini G., Amatiste S. (2018). Microbiological and chemical characteristics of pasta filata type cheese from raw ewe milk, using thermophilic and mesophilic starters. J. Food Saf. Food Qual..

[12] Verruck S., Dantas A., Schwinden Prudencio E. (2019). Functionality of the components from goat’s milk, recent advances for functional dairy products development and its implications on human health. J. Funct. Foods.

[13] Gonzales-Barron U., Gonçalves-Tenório A., Rodrigues V., Cadavez V. (2017). Foodborne pathogens in raw milk and cheese of sheep and goat origin: A meta-analysis approach. Curr. Opin. Food Sci..

[14] Trevisani E., Mancusi R., Valero A. (2014). Thermal inactivation kinetics of Shiga toxin-producing *Escherichia coli* in buffalo mozzarella curd. J. Dairy Sci..

[15] Tirloni E., Bernardi C., Rosshaug P.S., Stella S. (2019). Potential growth of *Listeria monocytogenes* in Italian mozzarella cheese as affected by microbiological and chemical-physical environment. J. Dairy Sci..

[16] Ustunol Z., Brown R.J. (1985). Effects of heat treatment and post treatment holding time on rennet clotting of milk. J. Dairy Sci..

[17] Shah R.D., Jana A., Solanky M.J. (2008). Use of plasticizing treatment in producing pasteurized Mozzarella cheese from raw milk. J. Food Sci. Technol. Mysore.

[18] Jana A., Mandal P.K. (2011). Manufacturing and quality of mozzarella cheese: A review. Int. J. Dairy Sci..

[19] IDF (1981). Milk: Determination of Fat Content. Butyrometer Gerber.

[20] ISO/IDF (2014). Milk and Milk Products—Determination of Nitrogen Content—Part 1: Kjeldahl Principle and Crude Protein Calculation.

[21] IDF (1986). Cheese and Processed Cheese Products: Determination of Dry Matter.

[22] IDF (1989). Determination of pH.

[23] ISO/IDF (2008). Cheese—Determination of fat content—Butyrometer for Van Gulik method.

[24] Trani A., Gambacorta G., Loizzo P., Cassone A., Fasciano C., Zambrini A.V., Faccia M. (2017). Comparison of HPLC-RI, LC/MS-MS and enzymatic assays for the analysis of residual lactose in lactose-free milk. Food Chem..

[25] Amagliani G., Petruzzelli A., Carloni E., Tonucci F., Foglini M., Micci E., Ricci M., Di Lullo S., Rotundo L., Brandi G. (2016). Presence of *Escherichia coli* O157, *Salmonella* spp., and *Listeria monocytogenes* in raw ovine milk destined for cheese production and evaluation of the equivalence between the analytical methods applied. Foodborne Pathog. Dis..

[26] Andrews A.T. (1983). Proteinases in normal bovine milk and their action on caseins. J. Dairy Res..

[27] Candiano G., Bruschi M., Musante L., Santucci L., Ghiggeri G.M., Carnemolla B., Orecchia P., Zardi L., Righetti P.G. (2004). Blue silver: A very sensitive colloidal Coomassie G-250 staining for proteome analysis. Electrophoresis.

[28] Trani A., Gambacorta G., Loizzo P., Alviti G., Schena A., Faccia M., Aquilanti L., Santarelli S., Di Luccia A. (2010). Biochemical traits of Ciauscolo, a spreadable typical Ttalian dry-cured sausage. J. Food Sci..

[29] Folch J., Lees M., Sloane-Stanley G.H. (1957). A simple method for isolation and purification of total lipids from animal tissues. J. Biol. Chem..

[30] Faccia M., Trani A., Natrella G., Gambacorta G. (2017). Chemical-sensory and volatile compound characterization of ricotta forte, a traditional fermented whey cheese. J. Dairy Sci..

[31] Calvo M.M. (2002). Influence of fat, heat treatments and species on milk rennet clotting properties and glycomacropeptide formation. Eur. Food Res. Technol..

[32] Kuo M.I., Gunasekaran S., Johnson M., Chen C. (2001). Nuclear magnetic resonance study of water mobility in pasta filata and non-pasta filata mozzarella. J. Dairy Sci..

[33] Sheehan J., Guinee T.P. (2004). Effect of pH and calcium level on the biochemical, textural and functional properties of reduced-fat Mozzarella cheese. Int. Dairy J..

[34] Law A.J.R., Leaver J. (1998). Effects of acidification and storage of milk on dissociation of bovine casein micelles. J. Agric. Food Chem..

[35] Altieri C., Scrocco C., Sinigaglia M., Del Nobile M.A. (2005). Use of chitosan to prolong mozzarella cheese shelf life. J. Dairy Sci..

[36] Faccia M., Gambacorta G., Natrella G., Caponio F. (2019). Shelf life extension of Italian mozzarella by use of calcium lactate buffered brine. Food Control..

[37] Ismail B., Nielsen S.S. (2010). Invited review: Plasmin protease in milk: Current knowledge and relevance to dairy industry. J. Dairy Sci..

[38] Kiely L.J., Kindstedt P.S., Hendricks G.M., Levis J.E., Yun J.J., Barbano D.M. (1993). Age related changes in the microstructure of mozzarella cheese. Food Struct..

[39] Faccia M., Trani A., Loizzo P., Gagliardi R., La Gatta B., Di Luccia A. (2014). Detection of αs1-I casein in mozzarella Fiordilatte: A possible tool to reveal the use of stored curd in cheesemaking. Food Control..

[40] Pesic M.B., Barac M.B., Stanojevic S.P., Ristic N.M., Macej O.D., Vrvic M.M. (2012). Heat induced casein–whey protein interactions at natural pH of milk: A comparison between caprine and bovine milk. Small Rum. Res..

[41] Murgia M.A., Deiana P., Nudda A., Correddu F., Montanari L., Mangia N.P. (2020). Assessment of microbiological quality and physicochemical parameters of Fruhe made by ovine and goat milk: A Sardinian (Italy) cheese. Fermentation.

[42] Ferrandini E., Castillo M., de Renobales M., Virto M.D., Garrido M.D., Rovira S., López M.B. (2012). Influence of lamb rennet paste on the lipolytic and sensory profile of Murcia al Vino cheese. J. Dairy Sci..

[43] Collins Y.F., McSweeney P.L.H., Wilkinson M.G. (2003). Lipolysis and free fatty acid catabolism in cheese: A review of current knowledge. Int. Dairy J..

[44] Mc Sweeney P.L.H. (2004). Biochemistry of cheese ripening. Int. J. Dairy Technol..

[45] Fedele V., Rubino R., Claps S., Sepe L., Morone G. (2005). Seasonal evolution of volatile compounds content and aromatic profile in milk and cheese from grazing goat. Small Rum. Res..

[46] Coppa M., Chassaing C., Sibra C., Cornu A., Verbič J., Golecký J., Engel E., Ratel J., Boudon A., Ferlay A. (2019). Forage system is the key driver of mountain milk specificity. J. Dairy Sci..

[47] Vasta V., D’Alessandro A.G., Priolo A., Petrotos K., Martemucci G. (2012). Volatile compound profile of ewe’s milk and meat of their suckling lambs in relation to pasture vs. indoor feeding system. Small Rum. Res..

[48] Ueda Y., Asakuma S., Miyaji M., Akiyama F. (2016). Effect of time at pasture and herbage intake on profile of volatile organic compounds of dairy cow milk. Anim. Sci. J..

[49] Narushima H., Omori T., Minoda Y. (1982). Microbial transformation of α-pinene. Eur. J. Appl. Microbiol. Biotechnol..

[50] Trudgill P.W. (1990). Microbial metabolism of monoterpenes—Recent developments. Biodegradation.

[51] Demyttenaere J.C.R., Atta-ur-Rahman (2001). Biotransformation of terpenoids by microorganisms. Studies in Natural Products Chemistry.

[52] Sgarbi E., Lazzi C., Tabanelli G., Gatti M., Neviani E., Gardini F. (2013). Non-starter lactic acid bacteria volatilomes produced using cheese components. J. Dairy Sci..

[53] Morales P., Fernández-García E., Nuñez M. (2005). Volatile compounds produced in cheese by *Pseudomonas* strains of dairy origin belonging to six different species. J. Agric. Food Chem..

